# Evidence against a contribution of the CCAAT-enhancer binding protein homologous protein (CHOP) in mediating neurotoxicity in rTg4510 mice

**DOI:** 10.1038/s41598-022-11025-x

**Published:** 2022-05-05

**Authors:** Marangelie Criado-Marrero, Danielle M. Blazier, Lauren A. Gould, Niat T. Gebru, Santiago Rodriguez Ospina, Debra S. Armendariz, April L. Darling, David Beaulieu-Abdelahad, Laura J. Blair

**Affiliations:** 1 Department of Molecular Medicine, Morsani College of Medicine, USF Health Byrd Alzheimer’s Institute, University of South Florida, Tampa, FL 33613 USA; 2 Research Service, James A Haley Veterans Hospital, 13000 Bruce B Downs Blvd, Tampa, FL 33612 USA

**Keywords:** Neuroscience, Diseases of the nervous system, Molecular neuroscience

## Abstract

Tau accumulation and progressive loss of neurons are associated with Alzheimer’s disease (AD). Aggregation of tau has been associated with endoplasmic reticulum (ER) stress and the activation of the unfolded protein response (UPR). While ER stress and the UPR have been linked to AD, the contribution of these pathways to tau-mediated neuronal death is still unknown. We tested the hypothesis that reducing C/EBP Homologous Protein (CHOP), a UPR induced transcription factor associated with cell death, would mitigate tau-mediated neurotoxicity through the ER stress pathway. To evaluate this, 8.5-month-old male rTg4510 tau transgenic mice were injected with a CHOP-targeting or scramble shRNA AAV9 that also expressed EGFP. Following behavioral assessment, brain tissue was collected at 12 months, when ER stress and neuronal loss is ongoing. No behavioral differences in locomotion, anxiety-like behavior, or learning and memory were found in shCHOP mice. Unexpectedly, mice expressing shCHOP had higher levels of CHOP, which did not affect neuronal count, UPR effector (ATF4), or tau tangles. Overall, this suggests that CHOP is a not a main contributor to neuronal death in rTg4510 mice. Taken together with previous studies, we conclude that ER stress, including CHOP upregulation, does not worsen outcomes in the tauopathic brain.

## Introduction

Cell adaptation to stress stimuli is essential to support neuronal health through our lifespan. Neurons, like other cells, need to respond and adapt quickly to harmful stimuli to ensure cell survival and prevent development of pathologies. The endoplasmic reticulum (ER) unfolded protein response (UPR) has an essential role in the cell to maintain homeostasis^1^. The UPR pathway is initiated by sensing the accumulation of misfolded proteins. UPR activation leads to the induction of molecular chaperones, slowed general translation, and initiation of degradation pathways including autophagy and ER-associated degradation (ERAD)^2^. The end goal of this process is to reduce the load of damaged proteins and restore cell homeostasis. Failure to complete this quality control favors the buildup of damaged proteins, which increases ER stress^3^.

Prolonged ER stress conditions can shift UPR pathway from a pro-survival to a pro-apoptotic state involving the induction of cell death proteins including CCAAT-enhancer binding protein (C/EBP) homologous protein (CHOP)^4–6^. UPR drives CHOP transcription by activating three upstream stress-sensing membrane proteins: Protein Kinase RNA-Like ER Kinase (PERK), activating transcription factor 6 (ATF6), and inositol-requiring protein 1 (IRE1α). PERK is the most characterized sensor protein, and like the others, it contains a cytoplasmic and ER luminal domain to transfer stress signal across the membrane^7^. Upon UPR activation, PERK dissociates from BiP (ER chaperone glucose regulated protein 78, Grp78), homodimerizes, and autophosphorylates (active form)^8^. Activated PERK prevents translation of proteins by the phosphorylation (inactivation) of the translation initiation factor, eIF2α, which then induces the activating transcription factor 4 (ATF4) followed by CHOP transcription^9,10^. Simultaneously, accumulation of misfolded proteins causes BiP to dissociate from ATF6 and IRE1α, which are activated after proteolytic cleavage and mRNA splicing, respectively^11–13^. Together, these UPR pathways continually respond to stress and decide the fate of the cells under normal or pathological conditions.

Recent evidence from in vitro and in vivo studies have linked ER stress to variety of neurodegenerative and age-related diseases, like Alzheimer’s disease (AD)^14–18^. The pathological accumulation of the microtubule associated protein tau has been observed in AD and has been correlated with ER stress markers^19^. Together with other studies, our lab showed increased signs of UPR activation in human AD and tau transgenic mouse brain areas such as the cortex and hippocampus^15,18–20^. The hippocampus is one of the first areas affected during AD pathogenesis^21^. It is a main center for learning and memory as well as stress adaptation processes. In conditions where stressors are not properly regulated, like prolonged metabolic stress, UPR is activated in the hippocampus resulting in increased phospho-tau levels, which may accelerate AD pathology^17^. While not consistent across all reports^22–24^, at least three tau transgenic mouse lines (rTg4510, 3xTg, and rTg21221) have increased ER stress-induced CHOP^25,26^. In the rTg4510 model, the line used in the current study, activation of UPR was accompanied by higher levels of ubiquitinated proteins and tau aggregation^19^. This activation was progressive and could be reversed by reducing tau levels. rTg4510 mice also show deficits in synaptic plasticity, learning and memory, and neuronal loss, which are common pathological hallmarks in AD^27^.

Interestingly, tau is not overexpressed in AD, nor is it found in the ER, rather it accumulates in the cytosol over time suggesting a problem with ER adaptive response and UPR mechanisms. It is known that proteins homeostasis, including ER molecular chaperones, declines with aging, which debilitates our resistance to stress and promotes cell death. For example, PERK mRNA is reduced while CHOP and caspase 12 are elevated in the brains of aged rats^28–30^. This suggests that, when compared to young animals, chronic activation of UPR during aging may be a route to cell death in tau-related diseases like AD. However, the extent of tau neurotoxicity mediated through the ER stress pathway is still unknown. A recent study showed that mice lacking CHOP were protected from ER stress and cell death^4,31,32^. Thus, we hypothesized that silencing CHOP in tau transgenic mice would block ER stress mediated neuronal death and reveal the contribution of this pathway to overall neurotoxicity. The main goal of this study was to evaluate this hypothesis by targeting CHOP in the hippocampi of rTg4510 mice using AAV delivered shRNAs. Cognitive function and histopathological changes, including neuronal loss, were evaluated in the brains of these mice.

## Results

### CHOP levels in the hippocampus of rTg4510 mice

**Figure 1:**
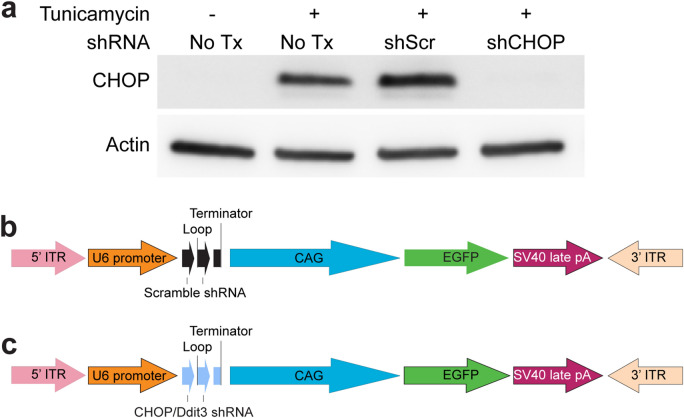
Validation of shRNA plasmid knockdown of CHOP in HT22 hippocampal cells. (**a**) Western blot of HT22 cell lysates that were transfected with non-targeting (Scramble) or CHOP-targeting shRNA for 72 h. To induce ER stress, tunicamycin was added to the media 24 h before harvesting. (**b**) Schematic of shScramble (shScr) and (**c**) shCHOP viral constructs. Ddit3, DNA Damage-Inducible Transcript 3—gene encoding CHOP. *CHOP* CCAAT-enhancer binding protein homologous protein, *EGFP* enhanced green fluorescent protein, *shRNA* short hairpin RNA, *Tx* treatment. Uncropped blots are provided in the Supplemental Information Fig. S1.

**Figure 2:**
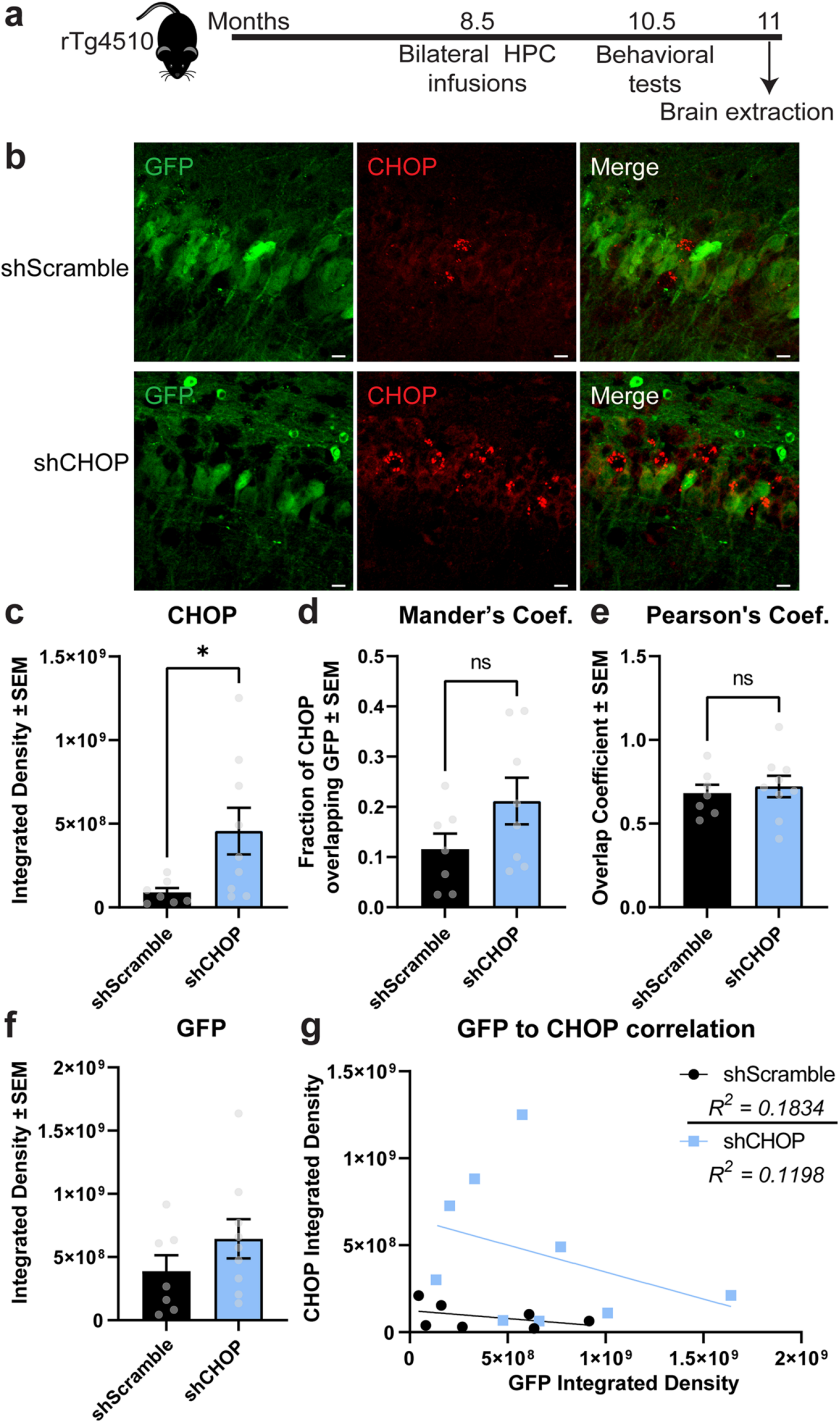
Experimental timeline and viral confirmation of shScramble and shCHOP injections. (**a**) rTg4510 mice received hippocampal injections of AAV9 shScramble or shCHOP containing EGFP tag at 8.5 months of age. (**b**) Representative images of EGFP and CHOP signal in hippocampal regions from mice expressing AAV9-shScramble [n = 7] or AAV9-shCHOP [n = 9]. (**c**) Sections expressing EGFP were analyzed for the integrated density of GADD153 (CHOP protein) staining in the hippocampus of rTg4510 mice injected as described above. Colocalization analysis was performed by (**d**) Mander’s coefficient (Coef.) to estimate the fraction of CHOP being colocalized with GFP and (**e**) Pearson’s Coef. to measure the co-occurrence of GFP/CHOP intensities. (**f**) Sections were also analyzed for the integrated density of GFP fluorescence in the hippocampus. (**g**) A correlation between viral transduction (GFP) and CHOP was performed. Data are represented in standard error of the mean (± SEM) and analyzed by unpaired Student’s t-test. Statistical significance is shown by *p = 0.0384. Scale bar represents 20 µm. *CHOP* CCAAT-enhancer binding protein homologous protein, *GFP* green fluorescent protein, *HPC* hippocampus.

Because elevated levels of CHOP have been linked to neurotoxicity and cell death^33^, we evaluated if the knockdown of this protein could mitigate neurotoxicity in the brains of tau transgenic mice. After identifying an shRNA that reduced CHOP levels in the HT22 mouse hippocampal neuronal cell line (Fig. 1a), we generated AAV9 virus (Fig. 1b,c) and injected the hippocampus of 8.5-month-old rTg4510 mice to express this construct (AAV9-shCHOP) and scramble control (AAV9-shScramble) (Fig. 2a). At this age, neuronal loss is already occurring, and ER stress markers are detectable in this mouse model^19,34,35^. The virus expressed for two and a half months before tissue collection. Upon investigation of AAV expression in these animals, GFP was nicely expressed (Fig. 2b), suggesting the AAV9 injections were successful. However, to our surprise, CHOP levels (also known as GADD153) were increased in the shCHOP injected mice (Fig. 2c), which may indicate the activation of UPR in these brains is a compensatory mechanism. To determine if the observed upregulation of CHOP occurred in cells neighboring those that were transduced (i.e. non-GFP), we quantified CHOP/GFP colocalization using both the Mander’s overlap coefficient (Fig. 2d) and Pearson correlation coefficient (Fig. 2e). No difference was found in the associations between CHOP and GFP between the groups. To confirm there was not a difference in viral transduction between the groups, we measured the GFP intensity density (Fig. 2f) and examined GFP/CHOP correlation (Fig. 2g). These analyses confirmed the GFP levels are similar in both groups and CHOP levels did not correlate to any mouse-to-mouse differences in GFP expression levels. This confirms that CHOP upregulation was not due to differences in viral infection efficiency. We also assessed CHOP levels in the entorhinal cortex (Fig. S2), which is an adjacent area to the hippocampus, to exclude the possibility that baseline levels were different between the injected groups, which confirmed CHOP levels did not differ between the mouse groups in a non-injected area. Therefore, CHOP levels are altered in the hippocampus of shCHOP injected mice.

### CHOP levels do not affect spatial cognition in tau transgenic mice

**Figure 3:**
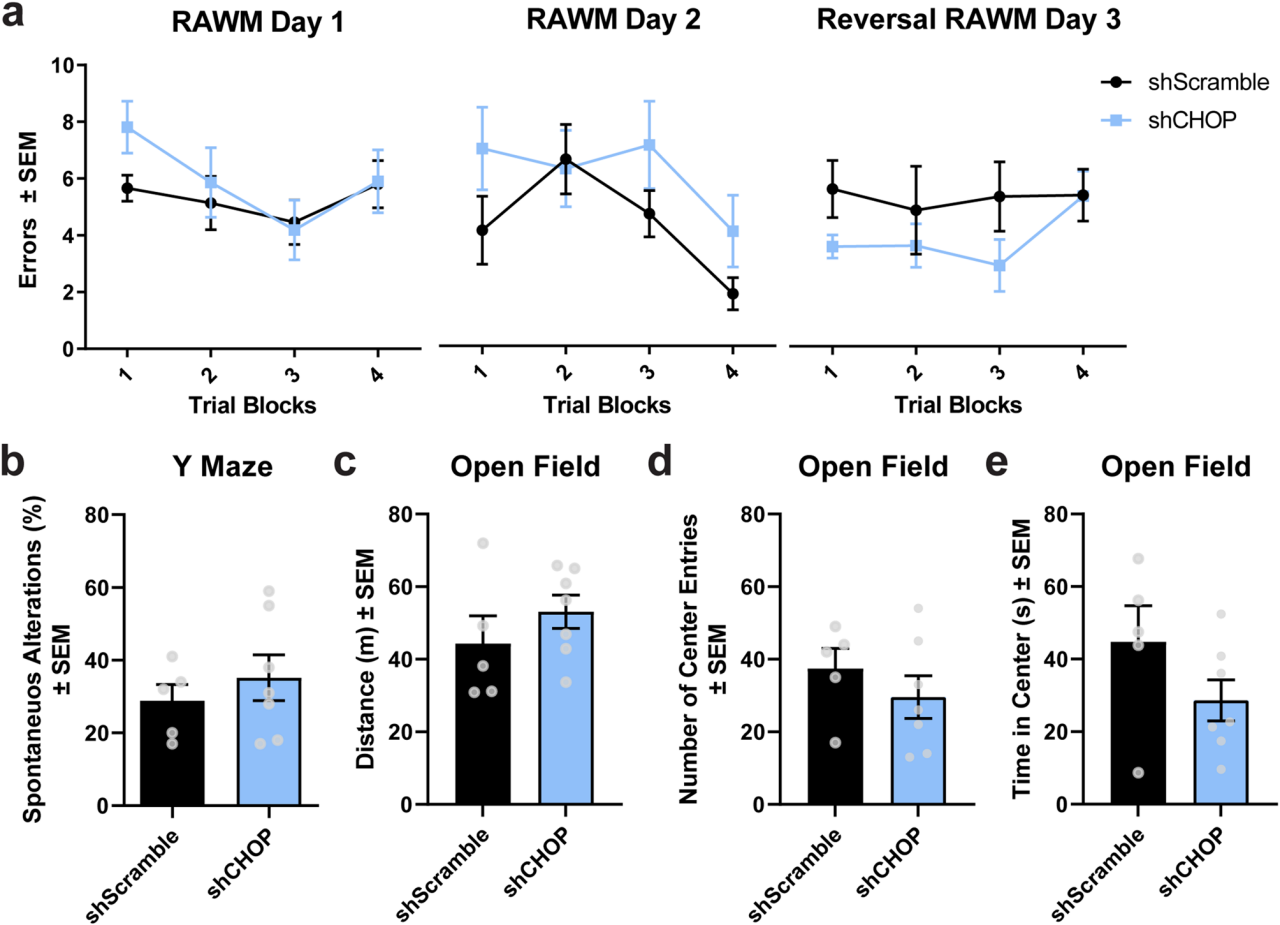
CHOP does not potentiate spatial memory deficits or anxiety-like behavior in rTg4510 mice. (**a**) Spatial memory was examined in using the radial arm water maze. (**b**) Y maze was used to assess working memory. (**c**) Locomotion (total distance travelled) and anxiety-like behavior by (**d**) number of entries and (**e**) time spent in the center area was measured using the open field test. A total of 12 rTg4510 mice [shScramble, n = 5; shCHOP, n = 7] were used for these tests. s = seconds; m = meters. Data is shown in standard error of the mean (± SEM) and analyzed by repeated measures one-way ANOVA (RAWM) or Student’s t-test (Y maze and open field).

Spatial learning and memory were not affected by altered CHOP levels in rTg4510 mice, as measured by the radial-arm water maze (RAWM) task (Fig. 3a). Spatial working memory, analyzed by the percentage of spontaneous alterations in the Y-maze, remained unchanged in shCHOP injected mice (Fig. 3b). Using the open field test, we confirmed that general motor activity (Fig. 3c) and anxiety-like behavior (Fig. 3d,e) was not affected. These data suggest that changes in hippocampal CHOP levels are not sufficient to modulate behavioral outcomes in these mice.

### Neuronal density is not altered after modulating CHOP in the hippocampus

**Figure 4:**
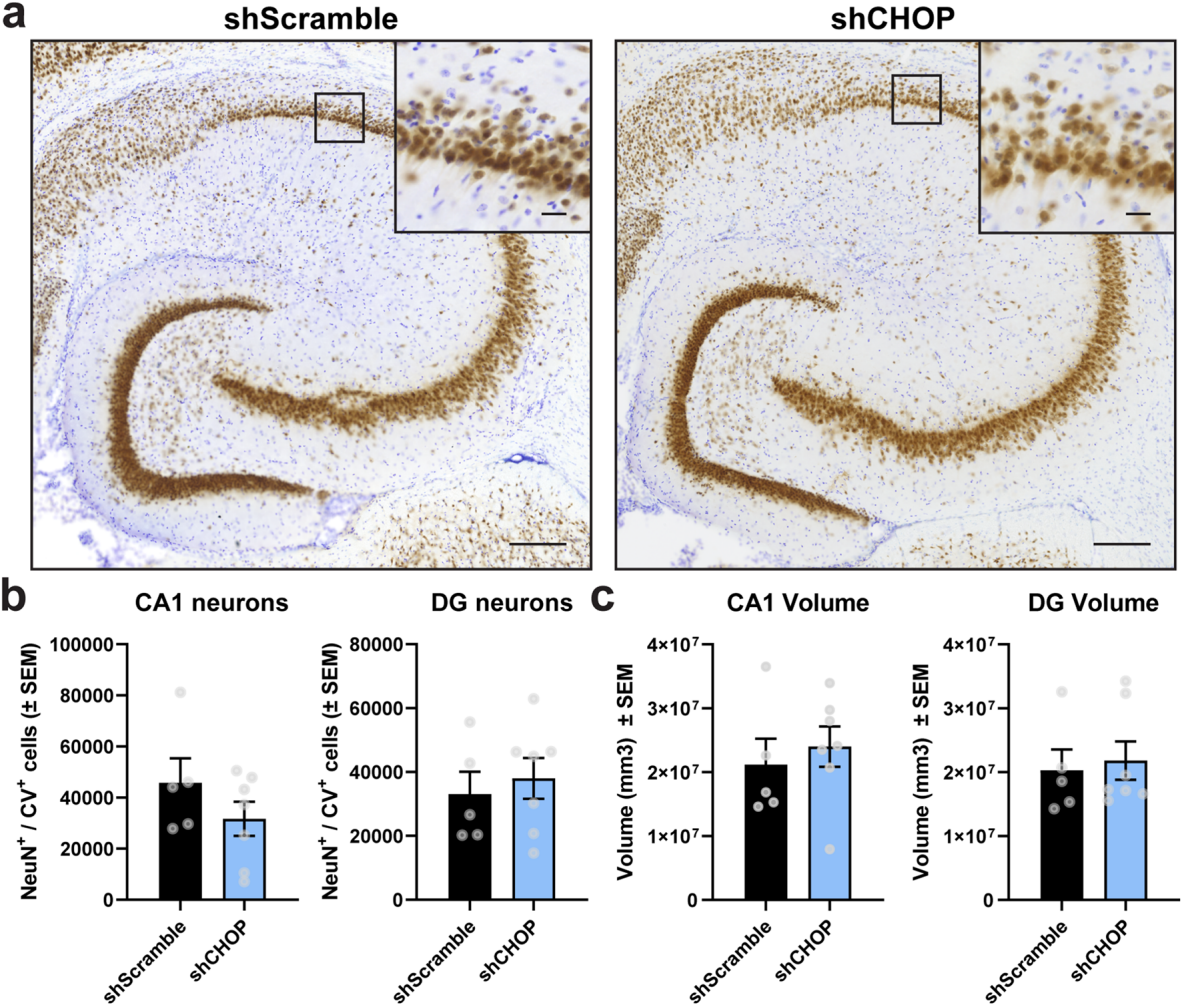
Changes in CHOP levels do not cause neuronal death in the hippocampus of aged rTg4510 mice. (**a**) Representative images from hippocampal neurons co-stained with NeuN (brown) and cresyl violet (purple) obtained from animals transduced with shScramble [n = 5] or shCHOP [n = 7] AAV9. (**b**) Neuronal density and (**c**) volume in the CA1 and DG areas were assessed using unbiased stereology. Results represent the standard error of the mean (± SEM). Data was analyzed by a Student’s t-test. Scale bars = 200 μm and inset scale represents 20 µm. *CV* cresyl violet, *CA1* Cornu ammonis subfield 1, *DG* dentate gyrus.

To determine whether neuronal loss was affected by altered CHOP levels, neuronal density was calculated using unbiased stereological analysis in which neurons positively stained for NeuN/cresyl violet in the CA1 and dentate gyrus hippocampal regions were counted (Fig. 4a,b). Both experimental groups showed similar number of neurons (Fig. 4b) and tissue volume in the areas analyzed (Fig. 4c), suggesting that changes in the CHOP pathway may not directly contribute to neuronal loss in rTg4510 mice.

### Gallyas-positive tau pathology does not decrease after modulating CHOP

**Figure 5:**
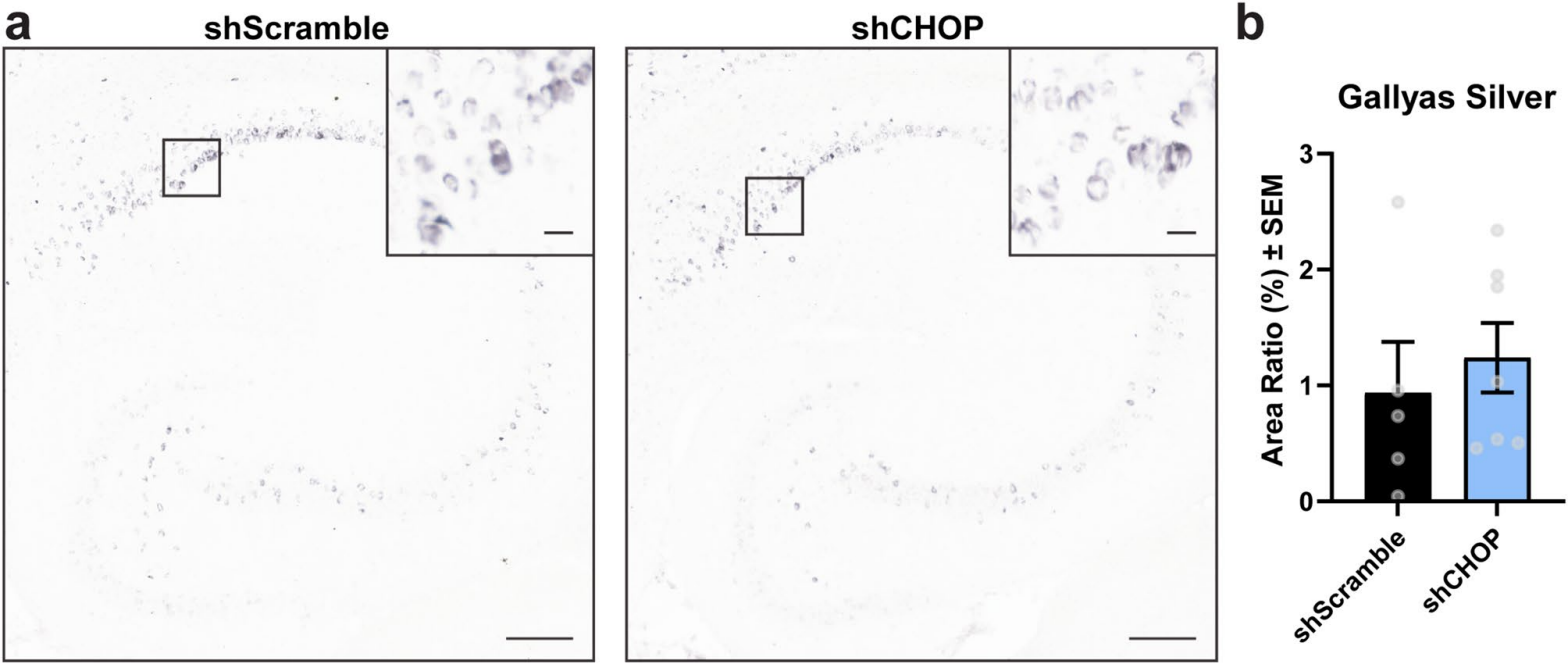
Tau tangles are not affected by CHOP. (**a**) Representative images and (**b**) analysis from hippocampal sections stained for Gallyas silver-positive tau in 11-month-old rTg4510 mice expressing AAV9-shScramble [n = 5] or AAV9-shCHOP [n = 7]. Area Ratio (%) was analyzed by unpaired Student’s t-test and results are represented as standard error of the mean (± SEM). Scale bar represents 200 µm; inset scale represents 20 µm.

The effect of CHOP levels on tau pathology was assessed by Gallyas silver staining. Here, the presence of argyrophilic tau remained unchanged when comparing hippocampal sections obtained from shScramble (control) to shCHOP injected rTg4510 mice (Fig. 5a,b). This shows that CHOP does not affect pre- and mature tau-like tangles in these mice.

### ATF4 levels are not significantly affected by CHOP induction in shCHOP-injected rTg4510 mice

**Figure 6:**
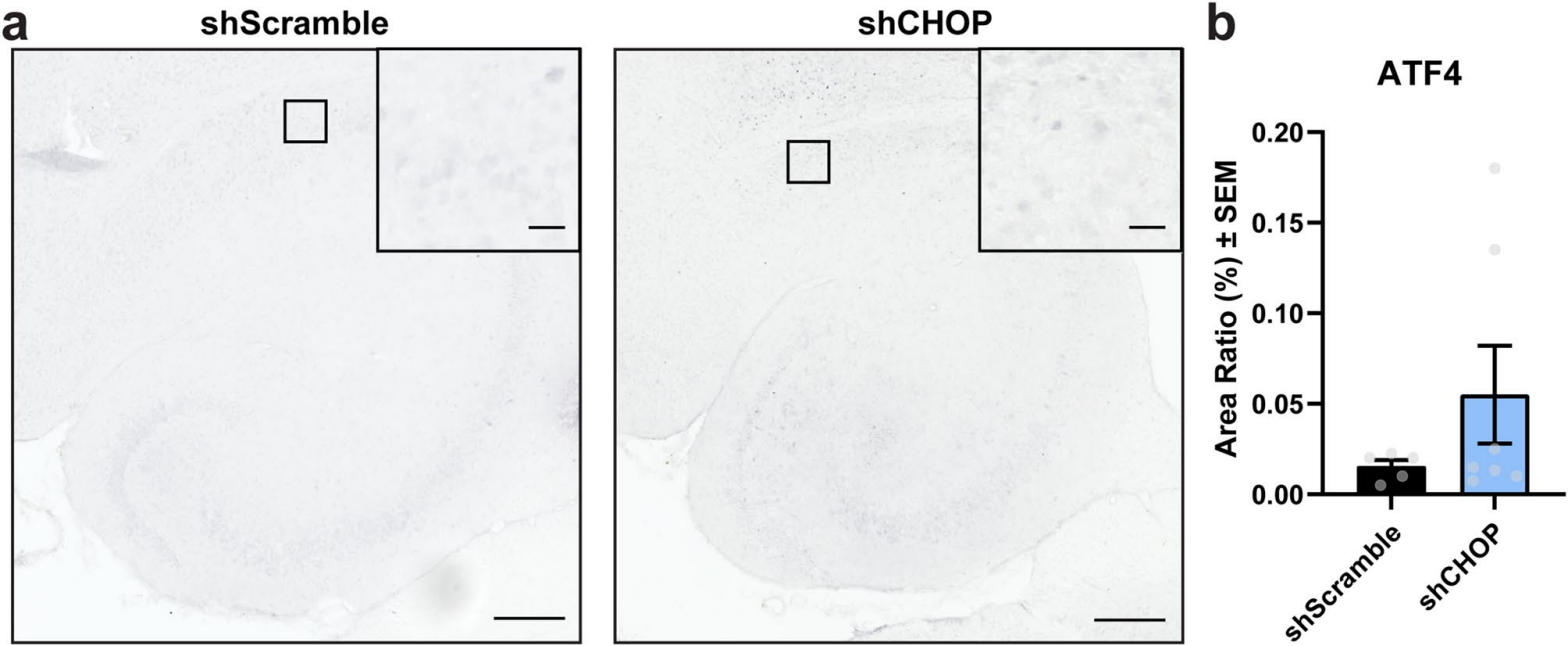
ATF4 levels are unchanged in shCHOP-injected mice. (**a**) Representative images and (**b**) analysis from hippocampal sections stained for ATF4 in 11-month-old rTg4510 mice expressing AAV9-shScramble [n = 5] or AAV9-shCHOP [n = 7]. Area Ratio (%) was analyzed by unpaired Student’s t-test and results are represented as standard error of the mean (± SEM). Scale bar represents 200 µm; inset scale represents 20 µm.

To determine if CHOP was being activated directly or as a result of increased UPR activation, we probed for ATF4 levels in the hippocampus of injected mice, since ATF4 is the transcription factor that induces CHOP in this pathway^9^. The levels of ATF4 levels in the hippocampal region was statistically comparable by Welch’s t-test between shCHOP and shScramble groups (Fig. 6a,b), which suggests the upregulation of CHOP levels was independent of ATF4 upregulation.

## Discussion

Here, we investigated whether CHOP, a downstream effector of ER stress, contributes to the neuronal loss and cognitive deficits observed in the rTg4510 tau transgenic mouse model^27,36^. We targeted CHOP in the hippocampus using AAV9 to express shRNA. Unexpectedly, we found higher levels of CHOP protein in the injected region suggesting a regulatory cellular feedback mechanism that led to increased transcription of CHOP. Spatial learning and memory, neuronal density, and tau positive tangles were not affected in these mice, indicating that altered CHOP levels do not contribute to these processes.

UPR activation has been detected in the brains of rTg4510 mice^19,26^. Because CHOP ablation has been previously demonstrated to block ER stress-mediated toxicity^37^, we anticipated the silencing of this pathway would decisively reveal the contribution to neuronal death in the brains of these mice. However, our outcomes were different than expected, because we found a compensatory upregulation of CHOP upon knockdown. This unintended upregulation of CHOP did not worsen neuronal toxicity or cognitive dysfunction, suggesting that this ER stress-induced factor may not be the main effector of declined neuronal health in this model. Like our observations, an earlier study showed that CHOP may not be a determining factor of cell death^38^. This is because to promote apoptosis CHOP may require additional factors, like ATF4 and GAAD34^38^. ATF4 levels were not affected in shCHOP when compared to control group possibly explaining the lack of effect on neuronal density. Another possible reason for this is that CHOP may be balancing between pro-survival and pro-apoptotic states^39,40^.

Based on numerous studies, CHOP may serve a mediator between autophagy and apoptosis during ER stress^39,41,42^. For example, low oxygen conditions upregulate CHOP which can bind to the promoter and increase transcription of Atg5, a key mediator of autophagosome formation^39^. During amino acid starvation, dual work of CHOP and PERK-induced ATF4 promotes expression of Atg5, p62, and Gaad34, which also serves as a modulator of autophagy^41,43^. p62 accumulation is linked to activation of autophagy for the removal of protein aggregates^44,45^. Also, p62 shuttles aggregated and ubiquitinated proteins like tau to ubiquitin-proteosome degradation^46^. We know that ubiquitin and p62 levels are increased in rTg4510 mouse and AD brains^19,20^, which may be mediated by CHOP upregulation. However, the lack of overall effect of CHOP in this study may be because its role is dependent on the timing of events and ER stress load^41,47^. A study from B’chir and colleagues showed that CHOP promotes autophagy during initial stages of starvation, while in later timings CHOP limited this process and initiated apoptosis and ER stress^41^. This is important because these findings suggest that under normal conditions CHOP may mediate cell survival by promoting autophagy pathways, but depending on the stress intensity and duration, it may switch to the induction of cell death genes.

Key cellular processes responsible for supporting protein homeostasis, like the UPR pathway, are known to decline with aging^48^. Likewise, transcription of p62 and other related autophagic genes decreases with aging augmenting the susceptibility to stress and debilitating the capability to degrade toxic proteins^49^. CHOP levels rise with age, which can induce other protein synthesis regulators (e.g. Gadd34), causing an environment with reduced ER chaperone function and poor stress response^28^. Similarly, UPR-related proteins like p-PERK are significantly increased in aged rTg4510 mice and AD brains^19,29^. This is interesting because higher levels of p-PERK appear to precede neurofibrillary tangles in AD and high p-PERK triggers degeneration of neurons via CHOP in mouse hippocampi^14,20,50–52^. Although our study did not assess impact of aging or exposure to different stressors, these factors will be highly relevant to understand the role of CHOP in age-related and neurodegenerative diseases.

Here, we investigated whether modulating CHOP expression in the hippocampi of rTg4510 affected Gallyas-positive tau inclusions. Measurements of toxic soluble tau species are missing in this study. Examining changes in tau aggregates, oligomers, and hyperphosphorylated species will be important to conclude whether the observed CHOP upregulation induced tau pathological changes. We showed that ATF4 was unaffected in shCHOP injected mice, however, including other proteins involved in the UPR signaling, like BiP, would be informative about the degree of activation of this pathway in neuronal health in this tau transgenic tau model. The lack of pathological changes in an environment where levels of CHOP are increased and ATF4 is unchanged, leaves an open pathway to explore in this model regarding whether both factors are needed to induce cell death in the brain. It is also important to note that AAV transduction has been previously shown to active the UPR, including the PERK and IRE1 pathways, which can trigger CHOP activation^53–55^. Without the direct comparison to AAV naïve mice, we cannot conclude if this contributed to our results. However, we do expect if AAV-mediated UPR activation did have any contribution that it would be similar between the groups based on their comparable GFP levels. We limited this study to the use of rTg4510 mice but investigating CHOP contribution in other tau transgenic models will be important to conclude whether CHOP mediates tau neurotoxicity. Our study suggests that future work should include crosses with CHOP knockout mice^31^ since knockdown of CHOP and the use of AAV may not give the desired effect. Finally, to better understand the contribution of CHOP and ER stress in neurodegeneration, further studies are needed to evaluate the contribution of sex differences, aging, damage to non-neuronal cells, and involvement in other pathways like neuroinflammation.

In conclusion, our results suggest that CHOP is not the main contributor to neuronal death in rTg4510 mice. CHOP may not be the primary player in rTg4510 neuronal death, but we cannot exclude the possibility of contributing to other processes like autophagy and protein translation. Thus, given its role in a variety of pathways and interplay with other cell regulatory factors, it may be important to examine CHOP’s function throughout AD pathological stages. Further studies could provide new insights for understanding whether CHOP plays a decisive role in protecting or driving cell death during the development of AD and other tauopathy-related neurodegenerative diseases.

## Methods

### Animals

Male mice were housed up to five per cage or individually when aggression was observed within the cage, which were acquired from the colony we maintained at the University of South Florida vivarium. Animals were kept in standard conditions with a 12-h light cycle (lights on at 6:00 and lights off at 18:00 h) while having free access to food and water. All experimental methods were performed following the National Institutes of Health Guide for the Care and Use of Laboratory Animals and were approved by the University of South Florida Institutional Animal Care and Use Committee (IACUC). All animal studies were conducted following the Animal Research: Reporting In Vivo Experiments (ARRIVE) guidelines. The sample size was based on prior studies with this strain of mice using intracerebral injections of AAV for similar types of analysis.

rTg4510 mice were obtained by crossing a Tg4510 line (FVB/N mouse genetic background containing a human MAPT P301L cDNA) and an activator line (mixed 129S6 and FVB/N mice genetic background expressing a tetracycline response element) under the control of a CaMKIIα promoter^34,56^. Since the tTA response element is controlled by a CaMKIIα promoter, tau is the restricted to excitatory neurons in the forebrain of the rTg4510 mice. Mice genotyping was performed after extracting DNA from ear tissue (2 mm punch) by a polymerase chain reaction (PCR) using the following primers: (1) Human Tau cd1F gene F [5’TGA ACC AGG ATG GCT GAG C 3′ and Tau cd5R, 5′TTG TCA TCG CTT CCA GTC C 3′], (2) tTA gene F [5′CGC TGT GGC ATT TTAC TTT AG 3′ and tTA gene R 5′CAT GTC CAG ATC GAA ATC GTC3’] and housekeeping gene T-cell receptor delta chain (Tcrd) [F, 5′-CAAATGTTGCTTGTCTGGTG-3′, and R, 5′-GTCAGTCGAGTGCACAGTTT-3′]. A QIAxcel Advanced system (Qiagen, Valencia, CA, USA) was used to analyze the DNA amplified PCR product.

### Validation of plasmids

HT22 cells, an immortalized mouse hippocampal cell line, were cultured and transfected for 72 h with non-targeting shRNA (Scramble, Sigma cat# SHC016) or CHOP/Ddit3-targeting shRNA (Sigma Mission library clone ID: TRCN0000324349) plasmids. Twenty-four hours before harvesting the cells, 10 µg/mL of tunicamycin was added to induce ER stress. Then, cells were collected for Western blot analysis. Membranes were probed overnight using CHOP/GADD153 (1:1000, Santa Cruz #sc-7351) and actin (1:1000, Sigma #A2066) antibodies.

### AAV production and purification

The short hairpin vectors targeting CHOP (shCHOP) or a control scramble sequence (shScramble) as validated above expressed the following RNA targeting sequence for shCHOP (GAAACGAAGAGGAAGAATCAACTCGAGTTGATTCTTCCTCTTCGTTTC) and for shScramble (CCTAAGGTTAAGTCGCCCTCGCTCGAGCGAGGGCGACTTAACCTTAGG). AAV9-shCHOP and AAV9-shScramble plasmids were designed and purchased from Vector Builder with a U6 promoter for the shRNA and a CAG promoter for an EGFP fluorescent tag. The production of AAV9 and purification was done in our lab as previously described^57^.

### Viral injections

At 8.5 months of age male rTg4510 mice were randomized to receive bilateral injections of AAV9 shCHOP or shScramble into the hippocampus (coordinates: X =  ± 3.6, Y =  − 3.5, and Z =  + 2.68) and cortex (coordinates: X =  ± 1.7, Y =  + 3.5, and Z =  + 2.7). Two microliters of 1 × 10^12^ viral particles/mL were delivered per injection site for each mouse using a robot stereotaxic drill and injection instrument (Neurostar GmbH, Tubingen, Germany).

### Tissue collection and staining

General locomotor activity and behavioral testing started eight weeks after viral injection. Following this, animals were anesthetized with Somnasol overdose and perfused with 0.9% saline at 11 months of age. Brains were collected and the left hemisphere was immersed overnight in 4% paraformaldehyde and transferred to gradient sucrose solutions (10, 20 and 30%) during the subsequent days. Using a sliding microtome, horizontal sections at 25 μm for immunofluorescence and Gallyas staining or at 50 μm for stereological analyses were obtained from these brains.

### Immunofluorescence and tissue staining

To detect CHOP expression, free floating sections including hippocampal area were stained as previously described^56^ using GADD153 (1:300, Novus Biological NBP213172) antibody followed by Alexa Fluor Goat α-Rabbit IgG-594 secondary antibody. Tissue was mounted onto slides and coverslipped with Prolong Gold Antifade reagent. ATF4 staining was performed using the primary ATF4/D4B8 (1:000, Cell Signaling 11815S) and anti-rabbit secondary (1:5000, Southern Biotech) antibodies. To prepare tissue for counting neuronal density, sections of 50 μm thickness were incubated with biotinylated NeuN antibody (1:3000, EMD Millipore MAB377B), mounted, and counterstained with 0.05% cresyl violet (Nissl) followed by dehydration with Xylenes and coverslipped with DPX solution (Sigma-Aldrich 06522). The Gallyas silver staining was used to detect tau positive neurofibrillary tangles as we have done previously^58^. Animals with low or poor expression of the virus in the hippocampal region were excluded in the analysis.

### Tissue imaging and quantification

Immunofluorescent staining for CHOP was imaged along with GFP expression using a 20× Plan-Apochromat 20× objective on a Zeiss LMS 880 AxioObserver confocal microscope. Slides stained for Gallyas and ATF4 were imaged using an Axio Scan.Z1 (ZEISS Microscopy, Munich, Germany) microscope slide scanner. Total Gallyas positive staining and ATF4 levels were determine using Nearcyte analysis software (Nearcyte.org). Regions of interest were outlined manually by a blinded investigator, and slides were analyzed by batch processing to determine the ratio of the positive staining area within the region of interest area (Area Ratio). The resulting Area Ratio was multiplied by 100 to generate Area Ratio (%). NeuN/cresyl violet positive neurons were counted using computer-based unbiased stereological analysis as we have done previously^58^. CHOP analysis was completed using the ImageJ/FIJI software. The “Try-it-all” option was used to set auto thresholds (16 possibilities) in all tissues. The three-best representation of thresholds among the tissue (low background and minimal non-specific signals) were averaged. We then analyzed GFP and CHOP integrated density by measuring the total sum of intensity units in each image, which was then averaged across all images for a single animal and then compared between injection groups. CHOP/GFP colocalization analysis was completed using the same program (JaCoP plugin in ImageJ/FIJI software) in the images showing CHOP upregulation. Mander’s quantification provides an estimate of the fraction of CHOP being colocalized with GFP while Pearson’s overlap coefficient indicates colocalization between GFP and CHOP intensities which ranges from 0 (null) to 1 (full). GraphPad PRISM software was used to calculate group mean differences between shCHOP and shScramble mice.

### Open field

Mice were acclimated to the testing room 1 h before all behavioral tests. These were all conducted during the morning by a blind experimenter to the study. Individual mice were placed in the center of the open field box, allowed to explore for 10 min, and video monitored using the Any-maze video tracking software (www.anymaze.com). Locomotion was evaluated in this test after calculating the total distance travelled. Anxiety-like symptoms were assessed by measuring the time spent in the center and number of entries to the center of the arena. The apparatus was clean with 10% ethanol after each trial.

### Y-maze

Short-term spatial memory was assessed by calculating the number of spontaneous alterations in the Y-maze. Mice were allowed to explore the 3-arm maze for a total of 8 min. Animal behavior was video tracked using the Any-maze software and analyzed by a blind experimenter to the study. Percentage of spontaneous alterations were counted as the total number of entries into each arm without any repeats minus two (alternative choices) and multiplied by 100.

### Radial-arm water maze (RAWM)

This behavioral task was used to evaluate spatial learning and memory performance as well cognitive flexibility in shScramble or shCHOP rTg4510 injected mice. The testing apparatus consisted of a water filled circular pool with 6-metal arms. Four different spatial cues were placed on the walls around the pool. There was an escape platform located at the end on one arm (defined as “goal arm”) and it was made visible using a raised flag or hidden (no flag). Animals were given 12 trials on each day and allowed to swim for up to 60 s to locate the platform. During training (day 1), the platform visibility was alternated with or without a flag between the trials. During memory test (day 2), the platform remained hidden throughout the 12 trials. To test their flexibility to learn, the platform was moved to the opposite direction of the goal arm. An entry to the incorrect arm (no-goal) or passing 15 s without arm entry was counted as an error. A blind experimenter to the study manually counted errors made on each trial.

### Statistical analysis

For the animal studies, the sample size was based on prior studies with this strain of mice using intracerebral injections of AAV for similar types of analysis. Normal distribution was examined by Shapiro-Wilks’s test. Using the GraphPad PRISM software version 9 (GraphPad Software, San Diego, CA, USA), differences between shScramble and shCHOP injected mice were determined by an unpaired Student t-test [Y maze, Open field, Stereology, Immunohistochemistry] and by two-way ANOVA [RAWM]. Outliers were identified using the Robust regression and Outlier removal (ROUT) method with PRISM software. Only mice lacking viral expression in the hippocampus were excluded in the study. Data is presented as the standard error of the mean (± SEM). Two-tailed p-values were calculated and considered significant when alpha level was equal or less than 0.05.

## Supplementary Information

Supplementary Figures.

## Data Availability

The datasets used and/or analyzed during the current study are available from the corresponding author on reasonable request.
