# Supplementary material for: Evidence against a contribution of the CCAAT-enhancer binding protein homologous protein (CHOP) in mediating neurotoxicity in rTg4510 mice
Source: Sci Rep. 2022 May 5;12:7372. doi: 10.1038/s41598-022-11025-x (PMC9072347; doi:10.1038/s41598-022-11025-x)
Supplement: Supplementary file 1 — Supplementary Figures. [file 41598_2022_11025_MOESM1_ESM.docx]

**Supplementary Information for**

**Evidence against a contribution of the CCAAT-enhancer binding protein homologous protein (CHOP) in mediating neurotoxicity in rTg4510 mice**

Marangelie Criado-Marrero^1^, Danielle M. Blazier^1^, Lauren A. Gould^1^, Niat T. Gebru^1^ Santiago Rodriguez Ospina^1^, Debra S. Armendariz^1^, April L. Darling^1^, David Beaulieu-Abdelahad^1^, Laura J. Blair^1,2,*^

^1^Department of Molecular Medicine, Morsani College of Medicine, USF Health Byrd Alzheimer's Institute, University of South Florida, Tampa, FL 33613, USA

^2^Research Service, James A Haley Veterans Hospital, 13000 Bruce B Downs Blvd, Tampa, FL 33612, USA

*Corresponding author

**Email:**  laurablair@usf.edu

ORCIDs. Laura Blair 0000-0002-4981-5564

**This PDF file includes:**

Supplementary material: Figure S1 and Figure S2.


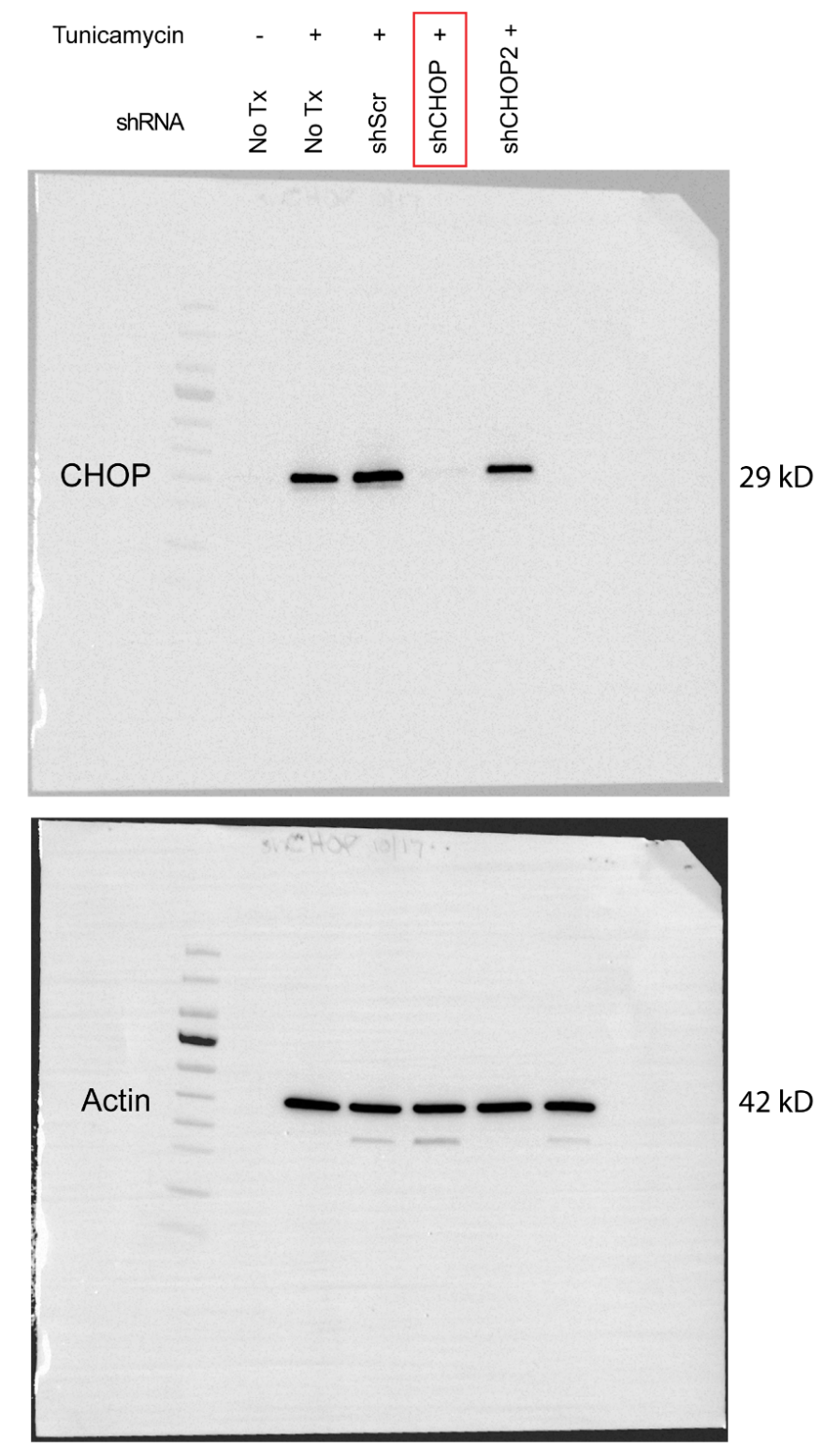


**Fig. S1. Western blot membranes showing CHOP knockdown in HT22 cells.** Western blot membranes of HT22 cell lysates incubated for 72 hours with shScramble (control) or two CHOP-targeting shRNAs (shCHOP and shCHOP2). Tunicamycin was added to the media 24 hours before harvesting the cells to induce ER stress. Membranes were probed for CHOP and Actin proteins to validate CHOP knockdown. Only shCHOP (indicated by red rectangle) was selected for in vivo studies because of its efficiency of knockdown CHOP protein.


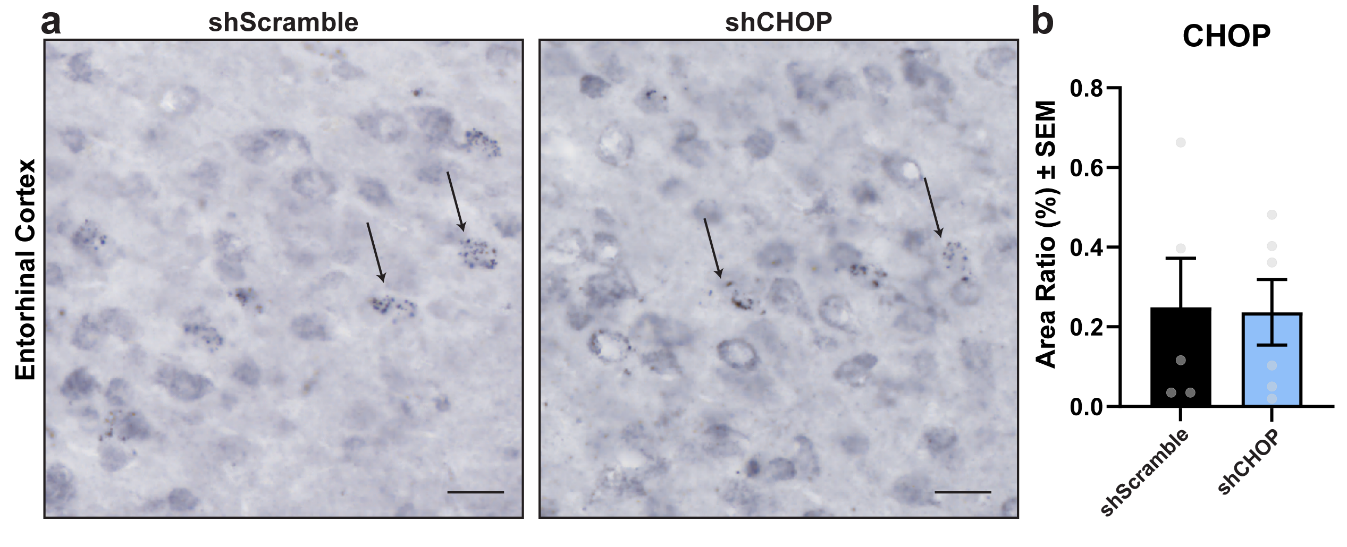


**Fig. S2. CHOP levels are unchanged in the entorhinal cortex (ECX).** **a** Representative images of CHOP staining in the entorhinal cortex (ECX) of rTg4510 mice expressing AAV9-shScramble or AAV9-shCHOP. **b** quantification of the Area Ratio (%) of CHOP protein levels in the ECX. Arrows indicate CHOP staining. Scale bar represents 20 µm. Results represent the standard error of the mean (± SEM). Data were analyzed by a Student’s t-test.
